# Problem behaviours and caregiver burden among children with Autism Spectrum Disorder in Kuching, Sarawak

**DOI:** 10.3389/fpsyt.2023.1244164

**Published:** 2023-10-30

**Authors:** Shi Yi Chua, Fairuz Nazri Abd Rahman, Selvasingam Ratnasingam

**Affiliations:** ^1^Department of Psychiatry, Faculty of Medicine, Universiti Kebangsaan Malaysia, Kuala Lumpur, Malaysia; ^2^Department of Psychiatry and Mental Health, Hospital Umum Sarawak, Kuching, Sarawak, Malaysia; ^3^Child and Adolescent Psychiatric Unit, UKM Specialist Children’s Hospital, Kuala Lumpur, Malaysia

**Keywords:** problem behaviours, caregiver burden, children, autism, cross-sectional study, Sarawak (Malaysia)

## Abstract

**Objective:**

Caregivers of children with Autism Spectrum Disorder (ASD) often experience emotional and psychological distress, as well as disruptions to family life and employment due to the challenges of caring for children with ASD. This study examines the relationship between problem behaviours and caregiver burden among children with ASD.

**Method:**

A cross-sectional study using convenience sampling recruited 230 caregivers of children with ASD aged 4 to 18 years from selected autism centres in Kuching, Sarawak. The caregivers completed the Aberrant Behaviour Checklist-2 and the Zarit Burden Interview.

**Results:**

Univariate analysis revealed a significant difference in caregiver burden for children with ASD receiving medications (*p* = 0.013), registered with the Social Welfare Department (*p* = 0.036), and having siblings with ASD (*p* = 0.046). About 40% of the children exhibited at least one domain of problem behaviour. More than half of the caregivers (53.9%) experienced burden, with the majority experiencing mild burden. Positive associations were seen between irritability (*r* = 0.458, *p* < 0.01), social withdrawal (*r* = 0.439, *p* < 0.01), stereotypic behaviour (*r* = 0.392, *p* < 0.01), hyperactivity/non-compliance (*r* = 0.467, *p* < 0.01), and caregiver burden. Child factors, including the duration of problem behaviour (*r* = 0.182, *p* = 0.007), medication use (eta = 0.187, *p* = 0.005), Social Welfare Department registration (eta = 0.138, *p* = 0.036), and the presence of siblings with ASD (eta = 0.130, *p* = 0.046) were associated with caregiver burden. Multiple linear regression showed that hyperactivity/noncompliance significantly predicted caregiver burden.

**Conclusion:**

Specific problem behaviours in children with ASD were associated with caregiver burden. These results highlight the need for interventions for the child with ASD and their caregivers.

## 1. Introduction

Autism Spectrum Disorder (ASD) is a neurodevelopmental disorder which is characterized by persistent deficits in social communication and social interaction as well as restricted, repetitive patterns of behaviour, interests or activities which causes clinically significant impairment in social, occupational, and other important areas of functioning (1). A feasibility study on the use of Modified Checklist for Autism in toddlers (M-CHAT) among children of 18 to 36 months of age in child health clinics by Ministry of Health Malaysia in 2006 showed that the prevalence of ASD in Malaysia was approximately 1.6 in 1000 (2).

Individuals with ASD experience a variety of emotional and behavioural problems that include externalizing and internalizing behaviours such as anxiety, depression, somatization, rule-breaking, aggression, self-harm, inattention, hyperactivity, impulsivity, and abnormal thought. Moreover, these symptoms change as the child grows and vary in severity (3). The prevalence of problem behaviour in children with ASD was found to range from around 71 to 94.0% (4–6). These figures are at least three times higher compared to an earlier study conducted in Malaysia, where the reported prevalence was considerably lower, at 24.2% (7). While problem behaviour was found to be independent of age and gender (6), several factors have been linked to problem behaviours in ASD, including having comorbid intellectual disability, impaired adaptive functioning, language impairment, as well as medical comorbidities such as seizures, gastrointestinal and sleep disorders (8–12). Moreover, except for hyperactivity, these behaviour problems tend to persist from adolescence through adulthood, indicating their chronic nature (11).

Problem behaviours affect both the child with ASD and their family members. Severe behaviour problems hamper the child’s development, learning, expression of adaptive behaviour and effectiveness of early intervention. It also results in more intensive use of medications (13) and an increased risk of hospitalization (14). In addition, problem behaviours adversely affect caregivers financially, psychologically, and physically (15–19). Prior research has demonstrated that siblings of children with ASD often undergo emotional distress due to problem behaviours, affecting their upbringing and their relationship with their siblings with ASD (20, 21).

Providing care for individuals with disabilities is associated with a variety of negative experiences (22). It is reported that caregiving for children with ASD is more stressful and challenging than parenting children with typical development and children with other developmental disabilities (23, 24). These challenges include behavioural problems, financial difficulties, stigma and the lack of awareness in the community (25). Problem behaviour has been one of the most widely reported predictors of caregiver stress and burden (3, 26–29). Other predictors of caregiver burden include the severity of ASD symptoms, lower levels of perceived social support, low financial status, caregiver parenting skills and caregivers having medical comorbidities (18, 30–32). There are several studies that attempt to examine the bidirectional relationship between child behaviour and caregiver stress within families of children with ASD. These studies suggest that child behaviour can exacerbate parental stress, and parental stress likewise exacerbates child behavioural problems (3, 33). Moreover, parent–child transactions were found to vary with different life phases and with different domains of behaviour problems (34).

Previous research has consistently demonstrated the significant impact of cultural differences on individuals’ perceptions of illness and their coping strategies during times of stress (35, 36). The majority of investigations examining the relationship between behavioural issues and caregiver burden have been conducted in Western and European countries (28, 37). These countries tend to embrace individualistic values, in contrast to Asian countries, that emphasize collectivism, where group interdependence and norms hold significance (38). In such collectivistic cultures, children with ASD displaying problem behaviours might be seen as devaluating the family’s reputation, leading to stigmatization within the family. Malaysia, particularly in Sarawak, boasts a diverse population consisting of Malay, Iban, Chinese, and Bidayuh ethnicities (39). Each of these ethnic groups maintains distinct cultural and spiritual beliefs that influence their perception of illnesses, health seeking behaviour and coping skills (40). Having a child with ASD may be associated with mystic beliefs or sometimes linked to ancestral past transgression, leading caregivers to seek spiritual treatment instead (41). The varying religious practices and teachings within Malaysia may also exert an influence on how caregivers navigate challenges (41–43). Consequently, this interplay between cultural beliefs, stigma, religious practices, and values can lead to differing perceptions of caregiver burden and stress when compared to other nations.

This study is aimed to examine the relationship between problem behaviours in children with ASD, sociodemographic variables, and caregiver burden in Sarawak, Malaysia. This would facilitate the delivery of locally tailored interventions for children with ASD and their caregivers. We hypothesize that there is a significant association between problem behaviours and caregiver burden in this population.

## 2. Methodology

### 2.1. Study setting, design, and participants

This is a cross-sectional study involving caregivers of children with ASD in the child & adolescent Psychiatric clinic and the child developmental clinic in Sarawak General Hospital, the Kuching Autism Association (KAA) headquarters and the One Stop Early Intervention Centre (OSEIC) in Kuching, Sarawak. Sarawak is geographically the largest state in Malaysia. It lies in East Malaysia and shares the island of Borneo with the eastern state of Sabah, Indonesian Kalimantan, and Brunei. It differs from other states in Malaysia in terms of socio-demographics particularly ethnicity, culture, religion, and socio-economic status. The child & adolescent Psychiatry clinic and the child developmental Clinic in Sarawak General Hospital cater mainly to children with autism in Kuching city. The Kuching Autism Association is a non-governmental organization which was established since 1998. The association runs an educational and pre-vocational training centre for children with ASD aged 3 to 17 years and a sheltered workshop for adults with ASD. The One Stop Early Intervention Centre (OSEIC) in Kuching is an early intervention centre which offers interventions for children with developmental or learning disabilities including ASD, Down Syndrome, and Intellectual Disability. It provides early intervention services which include physiotherapy, occupational therapy or speech therapy to infants and children up to the age of 6 years.

The recruitment of caregivers of children with ASD was done through convenient sampling. The data collection period was from 1st December 2021 to 31st March 2022. The inclusion criteria for the caregiver were those aged 18 years and above who provided unpaid care to the children for at least 12 months, have working knowledge of Malay or English, and were able to give consent for the study. Exclusion criteria were caregivers who took care of the children with ASD for less than 12 months and caregivers who were receiving any form of payment for their services. Inclusion criteria for the children are children who were aged between 4 to 18 years and diagnosed with ASD by a paediatrician or a psychiatrist based on the diagnostic criteria of DSM 5 or the International Classification of Disease 10 (ICD-10) and were taken care of by caregivers in the family context.

### 2.2. Data collection

The study population consisted of 230 caregivers of children with ASD visiting the above-mentioned centres in Kuching during the recruitment study period who fulfilled the inclusion criteria. All caregivers were briefed regarding the purpose of the study before consenting to participate in this study. Those who did not consent to the study were excluded from the study. Caregivers who agreed were requested to sign a consent before being enrolled in the study.

Each caregiver was given a set of 4 self-rating questionnaires which required about 20–25 min to complete. The caregivers were given explanations about the important instructions in each section of the questionnaires. The questionnaires were in English and Malay language. Caregivers were allowed to choose between the English or Malay-translated version of the questionnaires. The investigator was present to clarify any questions the caregivers had. Confidentiality was guaranteed to all participants. A coding system was used to organize the completed questionnaires. Caregivers and patients are not directly identifiable from the forms for the sake of confidentiality.

### 2.3. Study instruments

Permission from the original authors for the questionnaires Aberrant Behaviour Checklist-2 (ABC-2) and Zarit Burden Interview (ZBI) was obtained prior to commencing this study.

#### 2.3.1. Socio-demographic questionnaire

Information about the caregivers, including age, gender, ethnic group, the highest level of education, occupation, marital status, relationship with the patient, number of households, total household income (in Ringgit Malaysia), duration of caregiving and total contact hours were obtained. Patients’ socio-demographic data included age, gender, ethnic group, current or the highest level of education, types of specific intervention received (occupational therapy, speech therapy and/or applied behaviour therapy), the age and duration of these specific interventions received, other psychiatric illness, other medical illness, and the number of hospitalization due to problem behaviour, registration with the Social Welfare Department (Person with Disability status), benefits received from the Social Welfare Department or any other entity, and the presence of other family members with ASD were also gathered.

#### 2.3.2. Aberrant behaviour checklist 2nd edition

The ABC-2 (44) is a 58-item symptom checklist for rating inappropriate and maladaptive behaviours of school-age children or adults with mental disabilities. Scores on the 58 items resolve into five subscales: (1) irritability, agitation (e.g., disruptive behaviour), (2) lethargy, social withdrawal (e.g., isolation or inactivity), (3) stereotypic behaviour (e.g., repetitive purposeless movements), (4) hyperactivity, non-compliance (e.g., disobedient, overactive), and (5) inappropriate speech (e.g., repetitive talking). Each item is rated 0 to 3, with higher scores indicating greater severity. The authors report that domain scores falling above the 80th percentile can be defined as clinically significant. Numerous studies have been published supporting the reliability and validity of the ABC (45–47). The ABC has also been validated for Autism Spectrum Disorder (48). Kaat et al.’s study (48) also supported the standard factor structure of the ABC and the ABC’s construct validity in toddlers. The ABC-2 had been translated into multiple languages including Bahasa Malaysia. As the Malay ABC-2 is not validated in our local setting, a pilot test was carried out on 69 independent respondents. In this study, the Malay version of ABC-2 showed good internal consistency with the Cronbach’s *α* coefficient of at least 0.7 and above (see Supplementary Material).

#### 2.3.3. Zarit burden interview

The ZBI (49) was used to assess the level of subjective burden experienced by the caregivers of children and adolescents with ASD. It is a 22-item instrument used for measuring the caregiver’s perceived burden of providing family care. It was developed for use with caregivers of persons with dementia, but it had been used to assess many different types of caregivers, including those caring for persons with chronic health problems, mental health problems, and parents of children with health problems or behavioural and developmental problems. The 22 items are assessed on a 5-point Likert scale, ranging from 0 = “never” to 4 = “nearly always.” Item scores are added up to give a total score ranging from 0 to 88, with higher scores indicating a higher subjective burden. A total score of 0–20 indicates little or no burden, 21–40 indicates mild to moderate burden, 41–60 indicates moderate to severe burden, and 61–88 indicates severe burden. The questions focus on major areas such as burden in the relationship, emotional well-being, social and family life, finances, and loss of control over one’s life. It is available in English and Malay. It has recently been validated in the Malay language (50). The internal consistency of the Malay version of ZBI is good with a high Cronbach’s alpha coefficient of 0.898 and split-half correlation coefficient of 0.912. A score of 22 was selected as the suitable cut-off score for the Malay ZBI scale in the local population based on the ROC curve with the area under the curve of 0.786 (CI 0.658–0.914, *p* = 0.001). The Malay ZBI’s sensitivity and specificity were 70.8 percent and 69.2 percent, with a score of 22. In this study, the ZBI showed good internal consistency with Cronbach’s *α* coefficient of 0.91.

### 2.4. Statistical analysis

Statistical software, IBM-SPSS 26.0 (Armonk 2019), licensed to UKM, was used to complete the data analysis to answer the research questions of this study. Data was checked and cleaned. Preliminary data screening was done for missing values or possible wrong data entry. Normality assessment was also done for the data before proceeding to the analysis. The skewness values for all variables indicate that the distribution was normal since the values lay between −1 and 1.

Descriptive analysis was conducted for all the sociodemographic characteristics. Frequency and valid percentages were used to demonstrate categorical data. Means and standard deviation were used to demonstrate continuous variables. The mean caregiver total burden scores between children and caregiver sociodemographic variables were also compared using Independent *t*-test and one-way Analysis of Variance (ANOVA). Descriptive analysis was done for the five subscales of the ABC-2 (irritability, social withdrawal, stereotypic behaviour, hyperactivity, and inappropriate speech) and the total scores of the ZBI. Correlation analysis was done to assess the relationship between problem behaviour in children with ASD and caregiver burden. Simple linear regression analysis was done to determine if problem behaviour would predict caregiver burden. The association between child and caregiver variables and total burden scores was calculated using Eta correlation analysis for categorical variables. Pearson correlation analysis was used for continuous variables. A multiple linear regression analysis was also carried out to study the impact of the variables on caregiver burden.

### 2.5. Ethical aspect

This research was approved by the UKM research ethics committee (UKM PPI/111/8/JEP-2021-527). It was also registered under the National Medical Research Register Malaysia (NMRR-21-1179-59667). Approval was also obtained from Kuching Autism Association and One Stop Early Intervention Centre.

The caregivers with severe caregiver burden who were depressed or anxious during the interview were offered a referral to the adult psychiatric outpatient clinic with their consent. Additionally, they were advised about facilities where they could get assistance.

## 3. Results

In this study, a total of 250 caregivers were approached. There was a total of 170 (73.9%) caregivers from the clinical group and 60 (23.1%) caregivers from the non-clinical group. 20 caregivers did not consent for the study (8 caregivers from the clinical group and 12 caregivers from the non-clinical group). We do not have information about the clinical features of the children and parents who did not participate. About 15.2% were female and 84.8% were male children with ASD (see Table 1). The mean age of the patients was 8.37 years, indicating patients involved in this study were, on average, around 8 years old. Most of the patients were Malay, 45.2%. The mean duration of problem behaviour among children having ASD was 4.23 years. More than half (67.0%) of the children did not have any other psychiatric disorders. Most children with ASD, i.e., 91.7%, did not have any other medical conditions. More than half of the children, 65.2%, were registered with the Social Welfare Department. In addition, there were only 4.3% of children with ASD who had other siblings with the disorder.

**Table 1:** Demographics of children and adolescents with ASD.

Demographic variables	Mean (SD)	*n* (%)
Gender		
Female		35 (15.2)
Male		195 (84.8)
Age (years)	8.37 (3.535)	
Ethnicity		
Malay		104 (45.2)
Chinese		61 (26.5)
Indian		1 (0.4)
Bidayuh		30 (13.0)
Iban		22 (9.6)
Others (Bisaya, Jawa, Kadazan, Kayan, Kenyah and Melanau)		12 (5.2)
Type of class/school attended by the child at present		
No formal education		20 (8.7)
Early intervention program		36 (15.7)
Kindergarten		40 (17.4)
Mainstream school		23 (10.0)
Special education school		18 (7.8)
Integrated special education program		89 (38.7)
Others (Home School, Vocational School)		4 (1.7)
Duration of problem behaviour (years)	4.23 (2.852)	
Services/Treatment received by the child:		
i. Received early intervention program		
No		103 (44.8)
Yes		127 (55.2)
ii. Received medications		
No		178 (77.4)
Yes		52 (22.6)
iii. Received occupational therapy		
No		42 (18.3)
Yes		188 (81.7)
iv. Received speech therapy		
No		66 (28.7)
Yes		164 (71.3)
v. Received applied behaviour analysis		
No		189 (82.2)
Yes		41 (17.8)
vi. Received individual psychotherapy (i.e., cognitive behavioural therapy)		
No		217 (94.3)
Yes		13 (5.7)
vii. Received usual clinic follow up		
No		33 (14.3)
Yes		197 (85.7)
viii. Received other forms of services/treatment		
No		226 (98.3)
Yes (Neurofeedback)		4 (1.7)
Comorbid psychiatric illnesses		
None		154 (67.0)
Intellectual disability		10 (4.3)
Attention deficit hyperactivity disorder		60 (26.1)
Attention deficit hyperactivity disorder & intellectual disability		4 (1.7)
Others (Dyslexia, Language Disorder)		2 (0.8)
Comorbid medical illnesses		
None		211 (91.7)
Asthma		5 (2.2)
Epilepsy		4 (1.7)
Eczema		3 (1.3)
Others (Endocrinological problems, Allergic Rhinitis, Nephrotic Syndrome, Hereditary Spastic Paraplegia, G6PD Deficiency, Urological problems)		7 (3.1)
Registration with Social Welfare Department		
No		80 (34.8)
Yes		150 (65.2)
Benefits/Financial support from governmental or non-governmental agencies		
No		120 (52.5)
Yes		110 (47.8)
Other siblings with ASD		
No		220 (95.7)
Yes		10 (4.3)
Previous hospitalizations (due to problem behaviour)		
No		225 (97.8)
Yes		5 (2.2)

Children demographic data are summarized in Table 1.

Approximately 79.6% of carers were female, and 20.4% were male, as shown in Table 2. The mean age of the caregiver was 39.16 years, indicating that they were, on average, approximately 39 years old. In terms of ethnicity, 43.0% of the caregivers were Malay and around half of the caregivers (52.2%) had a tertiary education. Around half of the caregivers (52.2%) of caregivers held full-time employment. In terms of the marital status of caregivers, 93.9% were married. The majority of the caregivers (75.5%) were the patients’ mothers. According to the Department of Statistics Malaysia (51), household income is classified into 3 different income classifications – B40, M40, and T20. B40 represents the bottom 40% (household income is below RM 4,850 per month), M40 represents the middle 40% (household income between RM 4,851 per to RM 10,960 per month), and T20 represents the top 20% of Malaysian household income (household income exceeds RM 10,960 a month). Many caregivers had monthly household incomes below RM 4,850 (44.3%).

**Table 2:** Demographics of caregivers of children and adolescents with ASD.

Demographic variables	Mean (SD)	*n* (%)
Age (years)	39.16 (7.938)	
Gender		
Female		183 (79.6)
Male		47 (20.4)
Ethnicity		
Malay		99 (43.0)
Chinese		59 (25.7)
Indian		1 (0.4)
Iban		25 (10.9)
Bidayuh		30 (13.0)
Others (Bisaya, Jawa, Kayan, Kenyah, Melanau, and Punjabi)		16 (7.0)
Level of education		
Primary		8 (3.5)
Secondary		102 (44.3)
Tertiary		120 (52.2)
Occupation		
Unemployed/Homemaker		80 (34.8)
Employed part-time		4 (1.7)
Employed full time		120 (52.2)
Self-employed		24 (10.4)
Pensioner		2 (0.9)
Marital status		
Single		5 (2.2)
Married		216 (93.9)
Divorced		5 (2.2)
Widowed		4 (1.7)
Relationship with patient		
Father		45 (19.6)
Mother		174 (75.5)
Grandparent		3 (1.3)
Sibling		2 (0.9)
Others		6 (2.6)
Number of members in one household (persons)	5.17 (1.646)	
Total household income (per month)		
Less than RM 4850 (B40)		102 (44.3)
RM 4850-RM 10960 (M40)		96 (41.7)
More than RM 10960 (T20)		32 (13.9)
Duration of caregiving (years)	8.14 (3.705)	
Total daily contact hours with the patient (hours per day)	16.86 (5.138)	

Caregiver demographic data are summarized in Table 2.

### 3.1. Comparison of mean total burden score between child’s demographic variables and caregiver demographic variables

Independent *t*-test and One-way ANOVA were conducted to compare the mean caregiver total burden score of the children’s sociodemographic variables (see Table 3). Caregivers of children with ASD who received medications had higher burden scores (M = 30.08, SD = 15.68) compared to those who did not receive medications (M = 24.00, SD = 12.75); *t* (71.841) = −2.559, *p* = 0.013. Similarly, caregivers of children with ASD registered with the Social Welfare Department had higher burden scores (M = 26.75, SD = 13.95) compared to those not registered (M = 22.79, SD = 12.821); *t* (228) = −2.111, *p* = 0.036. Caregivers of children with ASD who had siblings with ASD also showed higher burden scores (M = 33.80, SD = 19.932) compared to those without siblings with ASD (M = 24.99, SD = 13.257); *t* (228) = 2.006, *p* = 0.046.

**Table 3:** Comparison of mean total burden score between child’s demographic variables.

Variable	*N*	Mean	Standard deviation	Independent *t*-test/1-way ANOVA	
				*t* statistic/*F* value	Value of *p*
Child gender^a^				−0.173	0.863
Female	35	25.740	13.507		
Male	195	25.310	13.735		
Ethnicity^b^				0.496	0.779
Malay	104	25.790	13.651		
Chinese	61	25.950	14.949		
Indian	1	39.000	0.000		
Bidayuh	30	23.900	12.729		
Iban	22	25.320	11.034		
Others (Bisaya, Jawa, Kadazan, Kayan, Kenyah and Melanau)	12	21.500	15.012		
Type of class/school attended by the child at present^b^				1.141	0.339
No formal education	20	24.900	14.563		
Early Intervention Program	36	26.860	14.090		
Kindergarten	40	24.380	14.181		
Mainstream School	23	19.870	9.474		
Special Education School	18	27.170	13.781		
Integrated Special Education Program	89	26.730	13.975		
Others (Home School, Vocational School)	4	17.750	10.340		
Services/treatment received by the child:					
i. Received early intervention program^a^				−1.160	0.250
No	103	24.210	14.044		
Yes	127	26.310	13.344		
ii. Received medications^a^				−2.559	0.013*
No	178	24.000	12.752		
Yes	52	30.080	15.679		
iii. Received occupational therapy^a^				−0.021	0.983
No	42	25.330	13.873		
Yes	188	25.380	13.664		
iv. Received speech therapy^a^				0.440	0.661
No	66	26.000	14.696		
Yes	164	25.120	13.277		
v. Received applied behaviour analysis^a^				−0.751	0.453
No	189	25.060	13.623		
Yes	41	26.830	13.975		
vi. Received individual psychotherapy (i.e., cognitive behavioural therapy)^a^				−0.149	0.882
No	217	25.340	13.640		
Yes	13	25.920	14.762		
vii. Received usual clinic follow up^a^				0.421	0.674
No	33	26.300	13.251		
Yes	197	25.220	13.768		
viii. Received other forms of services/treatment^a^				0.460	0.646
No	226	25.430	13.762		
Yes	4	22.250	7.136		
Comorbid psychiatric illnesses^b^				0.797	0.528
None	154	24.720	13.497		
Intellectual disability	10	26.300	11.186		
Attention deficit hyperactivity disorder	60	26.780	14.551		
Attention deficit hyperactivity disorder & intellectual disability	4	32.250	15.370		
Others (Dyslexia, Language disorder)	2	15.000	2.828		
Comorbid medical illnesses^b^				2.056	0.088
None	211	25.440	13.767		
Asthma	5	14.000	5.831		
Epilepsy	5	33.600	12.054		
Eczema	3	36.330	13.650		
Others (Endocrinological problems, allergic rhinitis, nephrotic syndrome, hereditary spastic paraplegia, g6pd deficiency, urological problems)	6	20.170	8.998		
Registration with social welfare department^a^				−2.111	0.036*
No	80	22.790	12.821		
Yes	150	26.750	13.951		
Benefits/Financial support from governmental or non-governmental agencies^a^				0.906	0.366
No	120	24.590	13.762		
Yes	110	26.230	13.584		
Presence of other siblings with ASD^a^				2.006	0.046
No	220	24.990	13.257		
Yes	10	33.800	19.932		
Previous hospitalizations (due to problem behaviour)^a^				−0.268	0.789
No	225	25.340	13.680		
Yes	5	27.000	14.782		

*Significant at <5% level.

a Independent *t*-test.

b 1-way ANOVA.

There were no significant difference in the mean total burden scores for the other children sociodemographic variables and caregivers’ sociodemographic variables.

### 3.2. Children with ASD having concerning levels of problem behaviour

In this sample, almost half (40.4%) of the children had at least one form of problem behaviour domain which had total scores above the 80th percentile (see Table 4). Around one-third (27.8%) had concerning levels of social withdrawal, followed by 22.2% of children having concerning levels of stereotypic behaviour, 18.3% of children having concerning levels of irritability, 13.9% of children having concerning levels of hyperactivity/non-compliance and 13.9% of children having concerning levels of inappropriate speech.

**Table 4:** Children with ASD with problem behaviour.

ABC-2 Variables	*n* (%)
Irritability	
Below 80th percentile	188 (81.7)
Above 80th percentile	42 (18.3)
Social withdrawal	
Below 80th percentile	166 (72.2)
Above 80th percentile	64 (27.8)
Stereotypic behaviour	
Below 80th percentile	179 (77.8)
Above 80th percentile	51 (22.2)
Hyperactivity/non-compliance	
Below 80th percentile	198 (86.1)
Above 80th percentile	32 (13.9)
Inappropriate speech	
Below 80th percentile	198 (86.1)
Above 80th percentile	32 (13.9)
Having at least one domain of problem behaviour above the 80th percentile	
None	137 (59.6)
Yes	93 (40.4)

### 3.3. Mean and standard deviation for ABC-2 subscales

The mean and standard deviation for the ABC-2 subscales are shown in Table 5.

**Table 5:** Mean and standard deviation for ABC-2 subscales.

ABC-2 subscales	Mean	Std. Deviation
Irritability	12.59	9.09
Social Withdrawal	12.33	8.12
Stereotypic Behaviour	5.57	4.82
Hyperactivity/Noncompliance	18.15	10.16
Inappropriate speech	3.18	2.72
Total	51.82	29.65

### 3.4. Total ZBI score and the level of burden among caregivers of children with ASD

The majority of the caregivers, 46.1%, were classified as having little or no burden, followed by 39.6% who were categorized as having mild to moderate level of burden, 13.5% who were classified as having moderate to severe burden and 0.9% with severe burden. Based on the results, the computed mean for the total ZBI score is 25.37 with a standard deviation of 13.672.

### 3.5. Correlation between problem behaviour subscales with caregiver burden

Pearson’s correlation analysis showed a significant positive correlation between the ABC-2 irritability, social withdrawal, stereotypic behaviour, and hyperactivity/noncompliance subscales with the total ZBI score. The irritability subscale showed a moderately positive correlation with the total ZBI score (*r* = 0.458, *p* < 0.01) (52). The Social Withdrawal subscale showed a moderately positive correlation with the total ZBI score (*r* = 0.439, *p* < 0.01). The Stereotypic Behaviour subscale showed a positive but weak correlation with the total ZBI score (*r* = 0.392, *p* < 0.01). The Hyperactivity/noncompliance subscale showed a positive moderate correlation with the total ZBI score (*r* = 0.467, *p* < 0.01). However, there was no association between the ABC-2 inappropriate speech subscale and the total ZBI score.

### 3.6. Relationship between sociodemographic variables and total burden scores

Correlation analysis showed a statistically significant association between the use of medication and total burden score (*p* < 0.05, eta = 0.187), indicating that there is a weak association between these two variables. In addition, the association between the duration of problem behaviour and total burden score was statistically significant (*p* < 0.05, *r* = 0.182), indicating that there is a weak association. There was also a statistically significant association between those registered with the Social Welfare Department and total burden score (*p* < 0.05, eta = 0.138), indicating a weak association between these two variables. Similarly, the association between the presence of other siblings with ASD and the total burden score was statistically significant (*p* < 0.05, eta = 0.130), indicating a weak association between the two variables. No other significant relationships were found with demographic variables.

### 3.7. Simple linear regression analysis

For Model 1 (as shown in Table 6), there is a significant positive relationship between Irritability and caregiver burden (value of *p* < 0.05, *B* = 0.688), with a 0.688 unit increase in caregiver burden for every unit increase of irritability. Similarly, for Model 2, there is a significant positive relationship between social withdrawal and caregiver burden (value of *p* < 0.05, *B* = 0.740), with a 0.740 unit increase in caregiver burden for every unit increase of social withdrawal. For Model 3, stereotypic behaviour significantly has a positive influence on caregiver burden (value of *p* < 0.05, *B* = 1.114). This indicates that every 1 level increase in stereotypic behaviour increases 1.114 caregiver burden. For Model 4, hyperactivity/non-compliance significantly has a positive influence on caregiver burden (value of *p* < 0.05, *B* = 0.628). This indicates that every 1 level increase in hyperactivity/non-compliance increases 0.628 caregiver burden. For Model 5, the findings reveal that there is no significant effect between inappropriate speech on caregiver burden (value of *p* > 0.05, *B* = 0.553).

**Table 6:** Simple linear regression analysis.

Model	Variables	Beta	Value of *p*	(95% confidence interval)	
				Lower bound	Upper bound
1	Irritability	0.688	<0.001*	0.514	0.863
2	Social Withdrawal	0.740	<0.001*	0.542	0.937
3	Stereotypic Behaviour	1.114	<0.001*	0.773	1.454
4	Hyperactivity/Noncompliance	0.628	<0.001*	0.473	0.784
5	Inappropriate speech	0.553	0.0096	−0.098	1.204

*Significant at 5% level. Dependent variable: total burden score.

### 3.8. Multiple linear regression analysis for ABC-2 scores in relation to ZBI scores among caregivers

Multiple linear regression for all five ABC-2 variables showed that only hyperactivity/ noncompliance subscale significantly predicts ZBI score. Every one score increase in the hyperactivity/noncompliance increases ZBI by 0.294 unit. Model 1 accounts for 22.3% of the variation in caregiver burden. In Model 2, the hyperactive/noncompliance domain remained a significant predictor for ZBI after further adjusted for confounders such as duration of problem behaviour, use of medications, registration with social welfare department and presence of other siblings with ASD. Every one score increase in hyperactive domain increases ZBI by 0.383 unit. Model 2 accounts for 25.6% of the variation in caregiver burden. No multicollinearity issue was detected as the VIF values for all predictors were <5.0. The results of the multiple linear regression are shown in Table 7.

**Table 7:** Multiple linear regression on ABC-2 scores in relation to ZBI scores among caregiver.

ABC-2 variables	Model 1		Model 2	
	*B* (95% CI)	VIF	*B* (95% CI)	VIF
Irritability	0.227 (−0.091, 0.545)	3.235	0.207 (−0.106, 0.520)	3.275
Social withdrawal	0.199 (−0.135, 0.533)	2.871	0.124 (−0.216, 0.463)	3.099
Stereotypic Behaviour	0.238 (−0.255, 0.731)	2.210	0.116 (−0.378, 0.611)	2.322
Hyperactivity/Noncompliance	0.294 (0.028, 0.560)^**^	2.853	0.383 (0.108, 0.658)^**^	3.175
Inappropriate speech	−0.317 (−0.937, 0.303)	1.133	−0.317 (−0.927, 0.293)	1.144
Adjusted *R*^2^	0.240		0.286	

*B*, adjusted regression coefficient; CI, confidence interval; VIF, variance inflation factor; ^**^, *p* < 0.05. Model 1: irritability, social withdrawal, stereotypic behaviour, hyperactivity/noncompliance, inappropriate speech. Model 2: model 1 further adjusted for sociodemographic factors including duration of challenging behaviour, use of medications, registration with social welfare department, presence of other siblings with ASD.

## 4. Discussion

This study aimed to examine the relationship between problem behaviour and caregiver burden. The result showed that nearly half of the children with ASD had at least one form of problem behaviour. More than half of the caregivers experienced caregiver burden, with the majority experiencing mild burden. Additionally, there were significant positive correlations between problem behaviour subscales (irritability, social withdrawal, stereotypic behaviour, and hyperactivity/noncompliance) and caregiver burden. Child variables such as duration of problem behaviour, use of medications, registration with the Social Welfare Department, and the presence of other siblings with ASD were associated with caregiver burden. Additionally, results from the multiple linear regression demonstrated that the hyperactivity/noncompliance subscale significantly predicted caregiver burden.

In this study, the mean age of the caregivers was 39 years. This is consistent with other previous studies done where the mean age of caregivers ranges from 38.91 to 42.5 (53–55). The latest demographic report for Malaysia shows that Malays comprise 57.9% of the population. In our study, 43% of the caregivers were Malay, which is lower than the national figure. This difference may be due to the significant presence of indigenous ethnic groups in Sarawak (56). Most of the caregivers were mothers of the child with ASD. This is consistent with other studies (54, 57). The male-to-female ratio in our study was found to be slightly higher at 5.5:1 as compared to the global estimate of 4.2:1 (58). Additionally, this study found a lower rate of psychiatric comorbidity compared to other studies previously done. Possible reasons include the low levels of mental health literacy and stigmatizing attitudes towards mental health problems among the public in Malaysia (59, 60). The stigma associated with psychiatric and neurodevelopmental disorders may possibly compel individuals to refrain from labeling their afflicted family members with disorders and under-reporting the severity of behavioural issues.

In our study, a higher prevalence of problem behaviour (40.4%) was found compared to Rzepecka et al.’s study (28%) (61). The higher prevalence found in our study could be due to the higher proportion of children with ASD in our sample compared to Rzepecka’s sample which consist of children with ASD and/or ID. Individuals with autism were found to have significantly higher levels of psychopathology than individuals with intellectual disability (62). The prevalence we found was lower compared to other studies (ranging from 72 to 93.7%) in children with ASD (4, 6, 63). This difference may be due to higher stigma and limited awareness about ASD and mental illness in Malaysia (59, 60, 64), causing caregivers to report fewer symptoms their child might be having.

Previous studies have shown that participants in clinical settings had higher ABC scores between subscales compared to those in non-clinical settings (26, 65–68). In this study, the majority of our participants were recruited from clinical settings (73.9%), with 23.1% originating from non-clinical settings. Mean scores obtained in our study differed from those in previous research on behavioural problems. Sannar et al. reported higher mean scores, likely because their study focused on children with Autism in a specialized inpatient psychiatric unit (66), who may have more severe behavioural issues than our participants who were drawn from outpatient clinics and the community. In contrast, our study found higher mean ABC-2 scores compared to a study by Kang et al. in Singapore (65). Kang et al. recruited their participants from an outpatient child developmental unit in a tertiary hospital, similar to our study. This difference could be because Singapore offers more accessible intervention services than Malaysia, resulting in fewer behavioural problems (69). In a Malaysian study examining the association between Vitamin D deficiency and behavioural symptoms in children with ASD, mean ABC-2 scores closely resembled our findings (70) with slightly elevated scores observed in irritability, social withdrawal, and hyperactivity/noncompliance subscales when compared to the group without Vitamin D deficiency at baseline. This may be due to the more similar demographic and cultural backgrounds of the participants in both studies.

In this study, most of the caregivers were found to have little or no burden. This is followed by caregivers who had mild to moderate burden. This suggests that the level of burden experienced by caregivers of children with ASD in this population was low. This is inconsistent with prior studies which found that perceived stress is moderate to high among caregivers of children with ASD (17, 23, 71–73). In Malaysia, cultural beliefs attribute having a child with ASD to past wrongdoings, and problem behaviours are perceived as bad parenting (36, 41, 64). Furthermore, individuals may view mental illness or stress as signs of weakness and incapacity (74). These prevailing beliefs and the associated stigma surrounding ASD and mental health issues can deter individuals from reporting symptoms due to the fear of judgment by others (64, 75). There are additional factors that could contribute to the lower reported burden in this study. Many studies have demonstrated the role of religious coping in dealing with stressful situations, physical and mental health challenges (35, 42, 76). In Malaysia, the major religions practiced are Islam, Christianity, and Buddhism, and their religious beliefs appear to play a significant role in helping caregivers interpret and cope positively with their child’s disability (41, 43, 64, 76). Their religious beliefs have made it easier for them to reach a stage of acceptance, giving them a sense of meaning while caring for their child with ASD (41, 76–78). However, further research is necessary to ascertain the extent to which these factors contribute to the lower perceived burden in this region.

A significant positive correlation was found between problem behaviours (irritability, social withdrawal, stereotypic behaviour and hyperactivity/non-compliance) and caregiver burden, indicating a higher level of these problem behaviours are associated with a higher caregiver burden. The results from this study are consistent with previous studies (79–83). Tomanik et al. found that mothers of children with autism reported the greatest stress when their children were more irritable, lethargic/socially withdrawn, hyperactive/non-compliant, unable to take care of themselves, and unable to communicate or interact with others (26). Similar to this study, the study failed to show a significant relationship between inappropriate speech and maternal stress. Multiple linear regression analysis showed that the hyperactivity/noncompliance domain remained a significant predictor for ZBI after further adjusted for covariates such as duration of problem behaviour, use of medications, registration with social welfare department and presence of other siblings with ASD. This supports previous research suggesting hyperactivity, noncompliance, aggression, and disruptive behaviours are significant predictors of parenting stress in children with ASD (27, 82, 84–86). Hyperactivity and disruptive behaviour are more likely to increase caregiver burden as these behaviours are more socially inappropriate. Aside from that, inappropriate speech was found to have no significant correlation with caregiver burden. Compared to externalizing behaviours, inappropriate speech may be socially less distressing and less disruptive.

A significant association was found between children with ASD receiving medications and caregiver burden. Those who receive medications are associated with greater age, more severe autism, more severe intellectual disability and increased scores in ABC (61, 87). In addition, children with ASD were reported to have higher levels of health care office visits and prescription drug use compared with children without ASD (88). It is probable that the usage of medicine indicates more severe behavioural issues, which would explain the higher burden scores and correlation between caregiver burden and medication use.

In addition, there was a significant association between the presence of other siblings with ASD in the family and caregiver burden. This is consistent with the findings of Orsmond et al., who reported that mothers caring for another child with a disability (in addition to the child with ASD) had greater levels of depression and anxiety symptoms (89). Having more than two children with chronic illness was also associated with caregivers’ burnout (90). The presence of another family member with developmental disabilities requiring specialized care will incur additional financial costs, and necessitate greater physical, emotional, and psychological support. This may possibly explain the significant association between these two variables.

This study found a significant association between the duration of problem behaviour in children with ASD and caregiver burden. There is no research that examines the association between the duration of problem behaviour and caregiver burden in children with ASD. However, a few studies showed a similar association between caregiver burden and duration of illness in other chronic illnesses in children (91–93). Previous studies also demonstrated a bidirectional relationship between behavioural problems and family stress, showing that it covaries across time (3, 33, 94–96). Ultimately, this relationship perpetuates both problem behaviour and caregiver burden.

There was a significant association between Social Welfare Department registration and total burden score. There are no previous studies that found a similar relationship between these two variables. Those registered with the Social Welfare Department are eligible for several services, including a monthly allowance, discounts on public transportation, fee waivers for medical services in government facilities and assistance with admission to special education schools. These services may alleviate the financial and psychological burdens experienced by caregivers. Caregivers are strongly encouraged to register their child with the Social Welfare Department to benefit from the services provided by the government. It is probable that those children with Social Welfare Department registration come from a lower socio-economic background. They may not have the privilege and access to early intervention programs, which may cause their symptoms to worsen. Consequently, they may require more interventional services, medical care, and admission to special education schools in comparison to individuals with less debilitating symptoms. Therefore, this could explain the significant difference and association between having Social Welfare Department registration and caregiver burden.

### 4.1. Limitations and strengths

The present study has several limitations. The first limitation of this study is that the causal relationship between problem behaviour and caregiver burden could not be determined due to the cross-sectional nature of this study. Longitudinal studies should be done to assess the causal relationship between behavioural problems and caregivers. Another limitation of this study is the sampling method used. Convenience sampling may give rise to sampling bias. The use of convenience sampling may result in sampling bias. Caregivers who volunteered for the study might have better awareness about ASD and have less burden, making them more likely to participate than those who declined. Those who did not participate may have children with more severe behaviour problems and higher burden. Additionally, this study did not assess several crucial factors. These include caregiver medical illnesses, psychiatric comorbidities, perceived social support, religious beliefs, and coping skills, all of which could potentially influence caregiver burden. This study also did not investigate sex distribution among caregivers and its effect on the caregiving burden. As most of the caregivers in this study were mothers of children with ASD, the results may not be generalized to fathers or other family members who are caregivers. Aside from that, this study also did not assess the severity of ASD which may also affect the caregiver burden. Assessment tools to assess the severity of ASD symptoms include Autism Diagnosis Interview-Revised (ADI-R), Autism Diagnostic Observation Schedule (ADOS), and Childhood Autism Rating Scale (CARS). These assessment tools require training for administration and a longer assessment time. As a result of these factors and time limitations, the severity of ASD symptoms was not evaluated. In addition, both problem behaviour and caregiver burden were assessed using self-reported questionnaires, which is another limitation of this study. There may be bias in this study’s findings due to inaccurate reporting by the caregivers. Also, the ABC-2 tool was not validated in this context. Hence, results should be interpreted with caution and further studies are needed to evaluate its validity.

One of the strengths of this study is achieving an adequate sample size of 230. This improved the power of the study. The use of validated questionnaire such as the Zarit Burden Interview was an additional strength of the study.

## 5. Conclusion

In conclusion, the current study found that there was a positive association between problem behaviours (irritability, social withdrawal, stereotypic behaviour, and hyperactivity/noncompliance) and caregiver burden. Inappropriate speech was not associated with caregiver burden. Other factors that were also found to be associated with caregiver burden in this sample included the duration of problem behaviour, use of medications, the presence of other siblings with ASD in the family and being registered with the Social Welfare Department. These results underline the significance of locally tailored interventions for both the child with ASD and their caregivers. Future research should investigate the efficacy of interventions developed to assist both the child and the caregiver.

## Data availability statement

The original contributions presented in the study are included in the article/Supplementary material, further inquiries can be directed to the corresponding author.

## Ethics statement

The studies involving humans were approved by University Kebangsaan Malaysia Research Ethics Committee and the National Medical Research Register Malaysia. The studies were conducted in accordance with the local legislation and institutional requirements. The participants provided their written informed consent to participate in this study.

## Author contributions

SC, FA, and SR contributed to conception and design of the study. SC organized the database, performed the statistical analysis, and wrote the first draft of the manuscript. All authors contributed to the article and approved the submitted version.
