# Supplementary material for: Problem behaviours and caregiver burden among children with Autism Spectrum Disorder in Kuching, Sarawak
Source: Front Psychiatry. 2023 Oct 30;14:1244164. doi: 10.3389/fpsyt.2023.1244164 (PMC10642943; doi:10.3389/fpsyt.2023.1244164)
Supplement: Supplementary file 1 [file Table_1.pdf]

Supplementary Material

Reliability Analysis for ABC-2

Variable	Cronbach's Alpha	No of Items
Irritability	0.919	15
Social Withdrawal	0.898	16
Stereotypic Behaviour	0.909	7
Hyperactivity/ Noncompliance	0.929	16
Inappropriate Speech	0.728	4
